# Celiac Disease and Risk of Atrial Fibrillation: A Meta-analysis and Systematic Review

**DOI:** 10.7759/cureus.6997

**Published:** 2020-02-14

**Authors:** Diego F Hidalgo, Boonphiphop Boonpheng, Lubna Nasr, Sehrish Sikandar, Jessica Hidalgo, Maria Intriago

**Affiliations:** 1 Geriatrics, Jackson Memorial Hospital, University of Miami, Miami, USA; 2 Internal Medicine, East Tennessee State University, Johnson City, USA; 3 Geriatrics, University of Miami Miller School of Medicine, Miami, USA; 4 Geriatrics, Miami VAHS GRECC Veterans Successful Aging for Frail Elders (VSAFE), Miami, USA; 5 Internal Medicine, San Francisco de Quito University, Quito, ECU; 6 Medicina, Universidad Espiritu Santo, Guayaquil, ECU

**Keywords:** celiac disease, atrial fibrillation, gluten sensitivity, inflammation

## Abstract

Introduction

Several studies have found celiac disease may be associated with a variety of cardiac manifestations. Atrial fibrillation (AF) is one of the most common arrhythmias that can cause significant morbidity. However, the risk of atrial fibrillation in patients with celiac disease according to epidemiological studies remains unclear. The aim of this meta-analysis study is to assess the risk of atrial fibrillation in patients diagnosed with celiac disease compared to controls.

Methods

A systematic literature review was conducted in MEDLINE, EMBASE, Cochrane databases from inception through December 2017 to identify studies that evaluated the risk of atrial fibrillation in patients with celiac disease. We included randomized controlled trial, cross sectional and cohort studies that reported the odds ratio, relative risk, hazard ratio, and standardized incidence ratio comparing the risk of developing atrial fibrillation among patients with celiac disease, versus patients without celiac disease as control. The Newcastle-Ottawa scale was used to determine the quality of the studies. Effect estimates from individual studies were extracted and combined using random-effect, generic inverse variance method of DerSimonian and Laird.

Results

Celiac disease is an autoimmune condition. This inflammatory state predisposes patients to develop AF. After a review of the literature, four observational studies with a total of 64,397 participants were enrolled. The association between celiac disease and increased risk of atrial fibrillation was significant, with a pooled OR of 1.38 (95% CI: 1.01-1.88). No publication bias as assessed by the funnel plots and Egger's regression asymmetry test with p = 0.54. However, the heterogeneity of the included studies was high (I2 = 96).

Conclusion

A significant association between celiac disease and risk of atrial fibrillation was reported in this study. There is a 38% increased risk of atrial fibrillation. Additional studies are needed to clarify the mechanistic link between atrial fibrillation and celiac disease. Some of the limitations of this study are that all were observational studies, some were medical registry-based and there was high heterogeneity between studies.

## Introduction

Celiac disease (CD) is an autoimmune, multisystemic and chronic disorder characterized by inflammation and villous atrophy in the small intestine in individuals with genetic susceptibility to gluten [1]. The incidence in the United States is approximately 1% (0.1 celiac patient per 1000 live births), similar to many countries in Europe [2,3].

Patients with CD may have intestinal and extraintestinal manifestations. Intestinal manifestations include malabsorption, abdominal distention and diarrhea. Some of the most common extraintestinal manifestations are characterized by fatigue, osteoporosis, reproductive, neuropsychiatric, immune disorders including diabetes mellitus, and malignant neoplasm of the gastrointestinal system [4-6].

Also, many studies have reported cardiovascular diseases in individuals with CD, such as cerebrovascular events, cardiomyopathy, ischemic heart disease and atrial fibrillation (AF) [7-9]. These cardiovascular disorders including AF could be related to inflammation and oxidative stress present in individuals with CD [10]. This has been proven by atrial electro-mechanic delay (EMD) as an early marker of AF and cardiac fibrosis seen in many biopsies [11-13].

AF is one of the most common arrhythmias that can cause significant morbidity. There is an estimation of 2.7-6.1 million people in the United States with AF [14]. However, the risk of AF in patients with CD according to epidemiological studies remains unclear.

The aim of this meta-analysis study is to assess the risk of atrial fibrillation in patients diagnosed with celiac disease compared to controls.

## Materials and methods

Search strategy

Two investigators (DH and BB) personally reviewed the databases, MEDLINE, EMBASE and Cochrane from inception through December 2017 to identify studies that evaluated the risk of atrial fibrillation in patients with celiac disease. The search strategy included terms for ‘atrial fibrillation, ‘supraventricular tachycardia’, ‘tachyarrhythmia’, ‘gluten enteropathy’, ‘gluten sensitive’ and ‘celiac disease’.

Selection criteria

Any study, in order to be selected for this meta-analysis, had to fulfill the following parameters:

- Randomized controlled trial (RCT), cohort (either prospective or retrospective), case-control study or cross-sectional study published as original study in the databases used. These studies should investigate the risk of developing AF in patients diagnosed with celiac disease.

- Odds ratios (OR), relative risk (RR), hazard ratio (HR), and standardized incidence ratio (SIR) with 95% confidence intervals (CIs).

- Subjects without celiac disease were used as comparators in cohort and cross-sectional study.

**Figure 1:**
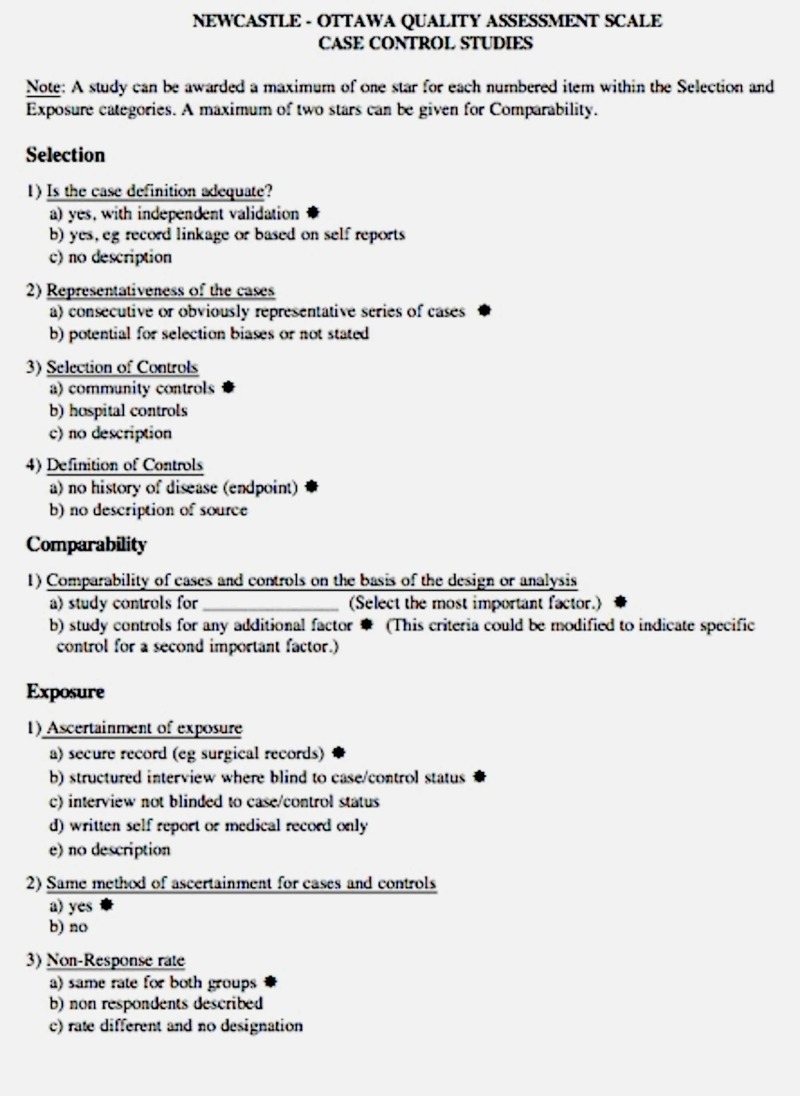
Newcastle-Ottawa quality assessment scale Adapted from [16].

In order to evaluate the quality of each study, the investigators independently used the validated Newcastle-Ottawa quality assessment scale (Figure 1). This scale evaluated each study in terms of selection of the participants, comparability between groups, as well as the ascertainment of the exposure of interest for case-control studies, and the outcome of interest for cohort studies [15].

Data extraction

A Microsoft Excel (Microsoft Corporation, Redmond, Washington, USA) data collection form was used to summarize the most relevant information obtained from these studies. This table contained the first author’s last name, the country where the study was conducted, year of publication, demographics, the Newcastle-Ottawa quality assessment scale, the total number of participants, characteristics of the participants, the method used to diagnose atrial fibrillation, the method used to determine celiac disease, adjusted effect estimates with 95% CI, and covariates that were adjusted in the multivariate analysis (Table 1).

**Table 1:** Characteristics of studies included CD: Celiac Disease; afib, AF: Atrial Fibrillation; GPRD: General Practice Research Database; DMII: Diabetes Mellitus Type 2; HTN: Hypertension; BMI: Body Mass Index; ICD: International Classification of Diseases.

	Lebwohl et al. [17]	Pattanshetty et al. [18]	Emilsson et al. [6]	West et al. [19]
Country	USA	USA	Sweden	United Kingdom
Study design	Cohort	Cohort	Cohort	Cohort
Year	2015	2014	2011	2004
Number of participants	7440	24530	28637	3790
Participants	Pathology reports from 28 pathology centers in Sweden between 2006-2008. Showing villous atrophy. Patients with CD who underwent more than one duodenal biopsy. CD patients who underwent follow-up biopsy between six months and five years after initial CD diagnosis.	Among a total of 22,385,340 patients, 24,530 were diagnosed with CD. The remaining 22,360,810 patients without CD served as the control group.	29,148 patients with CD through computerized biopsy reports from all of the 28 Swedish pathology departments. The biopsies were obtained from 1968 to 2008. A total of 459 patients were excluded as they developed afib before the time of the biopsy.	All subjects within the General Practice Research Database between June 1987 and April 2002 with a recorded diagnosis of coeliac disease aged 25 or older at the start of their GPRD record.
Mean age of participants in years	NA. 46% were between 0-19	NA	30	NA
Percentage of female	64%	61%	62.2%	68.6%
Diagnosis of afib	NA	NA	Diagnosis of AF: according to relevant ICD codes in the Swedish National Patient Register (Discharge diagnoses) and the Cause of Death Register, the definition of AF included inpatients and outpatients, as well as individuals diagnosed with AF as a cause of death. We included primary and secondary diagnosis of AF from the Swedish Patient Register, but only AFs that were listed as the main underlying cause of death.	Records review from the GPRD that included atrial fibrillation as a diagnosis after Celiac disease was diagnosed.
Diagnosis of celiac disease	Small intestine biopsy showing villous atrophy	NA	Intestinal biopsy reports (defined as Marsh 3: villous atrophy) from all pathology departments (n. 28) in Sweden.	Records review from the GPRD that included the diagnosis of coeliac disease aged 25 or older.
Adjusted OR or HR or IRR	Adjusted HR 0.97; 95% CI 0.73-1.30)	Odds ratio being 2.04 (95% CI 1.9 to 2.2). P < 0.001	Adjusted HR for AF was 1.34 (95% CI 1.24–1.44). P < 0.001	Adjusted odds ratio 1.26 (95% confidence interval: 0.97–1.64)
Confounder adjustment	Age, gender, duration of CD, calendar period, educational attainment.	Sex, age, race, DMII, HTN, Smoker.	Education, country of birth (Nordic country vs. not Nordic country), type 1 diabetes, autoimmune thyroid disease, or rheumatoid arthritis, birth weight, BMI, antihypertensive medication.	Height, weight, smoking, BMI, diabetes type II, thyroid disease.
Quality assessment (Newcastle-Ottawa scale)	Selection: 3, Comparability: 1, Outcome: 3	Selection: 4, Comparability: 1, Outcome: 2	Selection: 4, Comparability: 1, Outcome: 2	Selection: 4, Comparability: 1, Outcome: 2

To ensure accuracy, all investigators performed the data extraction process independently. Any data discrepancy was also resolved by referring back to the original article.

Statistical analysis

Data analysis was performed using Review Manager 5.3 software from the Cochrane Collaboration (London, United Kingdom). Adjusted point estimates and standard errors from the individual studies were combined using the generic inverse variance method of DerSimonian and Laird, which assigned the weight of each study based on its variance [20]. In light of the possible high between-study variance due to different study designs and populations, we used a random-effect model rather than a fixed-effect model. Cochran's Q test and I2 statistic were used to determine the between-study heterogeneity. A value of I2 of 0%-25% represents insignificant heterogeneity, greater than 25% but less than or equal to 50% represents low heterogeneity, greater than 50% but less than or equal to 75% represents moderate heterogeneity, and greater than 75% represents high heterogeneity [21].

## Results

**Figure 2:**
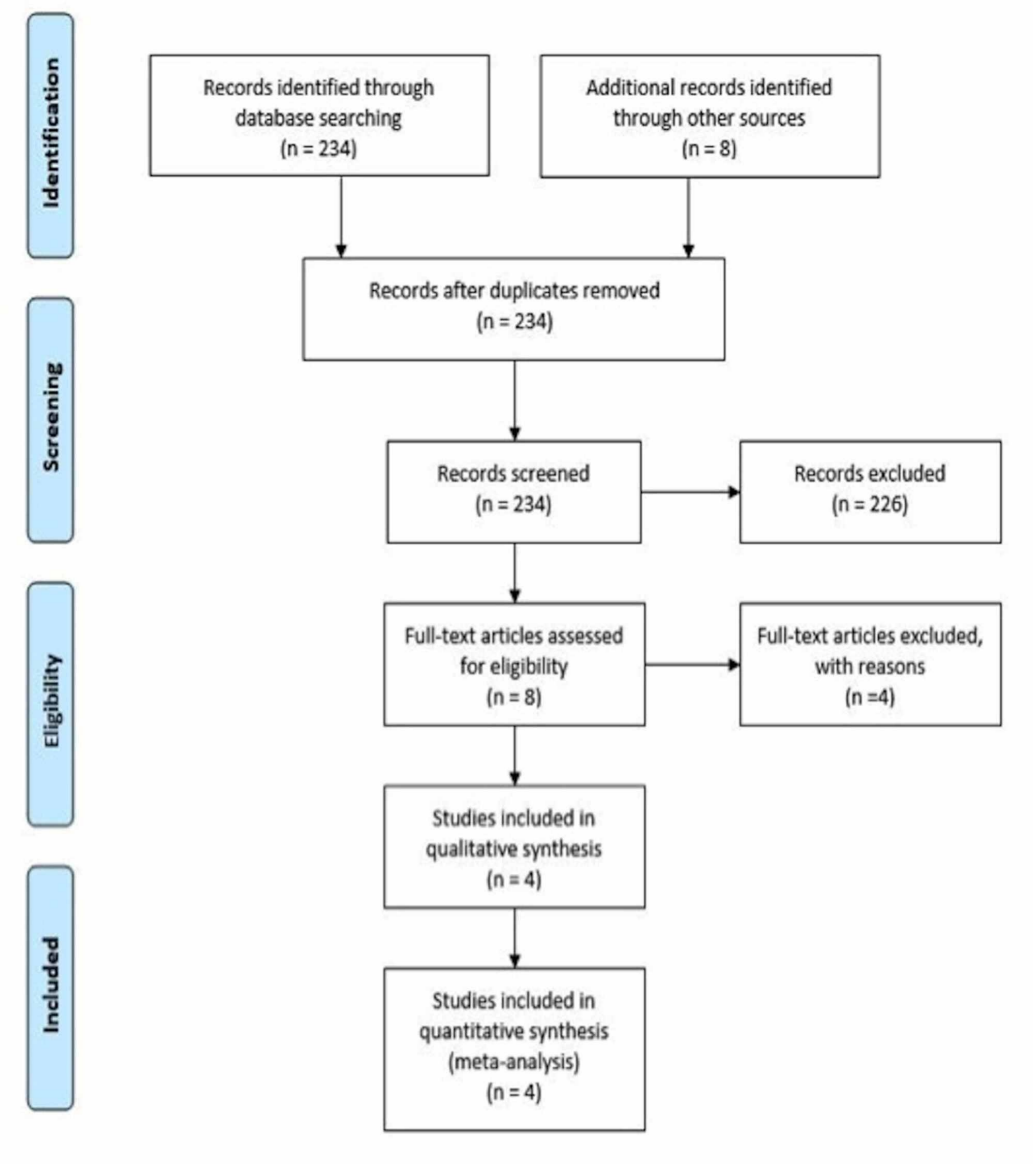
Search criteria and eligibility.

An advanced search yielded 234 articles on the databases. Eight additional articles were identified through other sources. After the exclusion of eight articles that were duplicated, 234 underwent a title and abstract review. A total of 226 articles were excluded, as they were case reports, book articles, letters to the editor, or review articles without the information needed for the analysis, leaving eight articles for a full-length article review. A total of four articles were dismissed at this time because they did not have comparators. A total of four studies were used for statistical analysis, all of them were cohort studies. The outlines of the literature review and study selection process are given in Figure 2. The clinical characteristics of each study and the quality assessment are described in Table 1.

**Figure 3:**
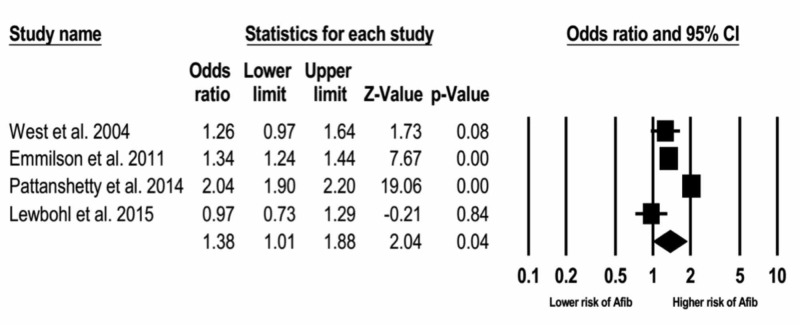
Relative risk and p-value.

The overall analysis found a higher risk of atrial fibrillation in patients with celiac disease as compared with the control individuals who did not have celiac disease. The odds ratio (OR) was 1.38 (95% CI: 1.01-1.88), p < 0.001, as shown in Figure 3.

To measure heterogeneity among the studies, Cochran’s Q test and I2 were calculated. The I2 calculated for this study was 96, which represents high heterogeneity among the studies.

**Figure 4:**
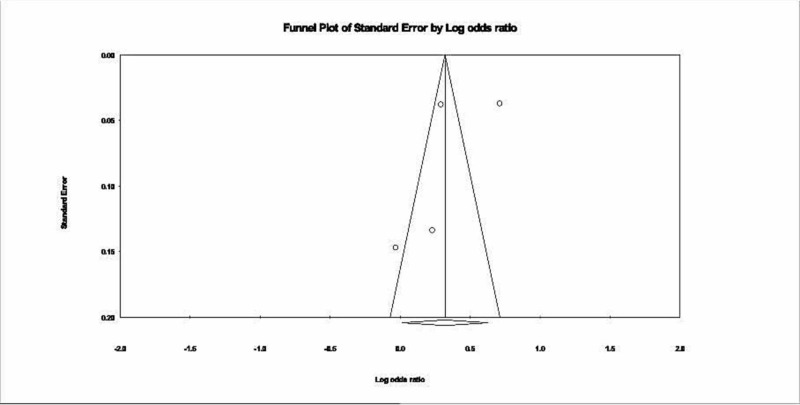
Publication bias, funnel plot.

The Egger regression test and funnel plots were used to assess publication bias. Egger’s regression test (P 0.54) did not show a publication bias. Funnel plots (Figure 4) were symmetrical, indicating low publication bias. The total number of studies was four, which correlates with adequate power for this test.

## Discussion

This meta-analysis study was performed to assess the risk of AF in patients diagnosed with CD. After the evaluation of four studies that met the inclusion criteria (cohort, case-control and cross sectional), with a total enrollment of 64,397 participants, we found that patients with CD have a 38% increased risk of developing AF. This study represents the first meta-analysis that assesses the relationship between CD and AF.

This study would be considered global research because it includes countries around the world such as USA, UK, Sweden, and others. It collects more than 60,000 patients and their records. Also, we included studies correlating the risk of AF in patients with CD confirmed by biopsy [22,23]. Emilsson et al. reported that patients having a later diagnosis of CD were at increased risk of AF (OR = 1.45; 95% CI = 1.31-1.62). The results of this study are similar than earlier studies performed worldwide [6]. In 2004, West et al. reported an increased risk of AF in CD (OR 1.26; 95% CI: 0.97-1.64) [18]. Both studies were similar to our findings (OR = 1.38; 95% CI: 1.01-1.88). Those results compared with controls, confirm that CD is associated with significantly increased risk of AF.

Also, many studies have supported the role of the immune system in the pathophysiology of AF in patients with CD. Inflammation and oxidative stress have been found to be responsible of many molecular mechanisms of CD including activation of immune cells such as macrophages, T and B cells, neutrophils and inflammatory cytokines (IL-6, TNF-α). These cytokines and activated immune cells could affect the contractility and electrical myocytes stability inducing fibroblast activation and cellular fibrosis [24-26]. These atrial changes provide reentrant arrhythmias confirmed clinically and electrocardiogram [27].

Regarding the strengths of this study, it includes research studies done all over the world, more than 60,000 patients were included and duplicated studies were removed. Among the limitations of this study: all were observational studies, some with medical registry-based, only four articles were included and there was high heterogeneity between these studies.

More studies should be done to determine the exact mechanism of how celiac disease increases the risk of atrial fibrillation. A good understanding of the mechanisms behind it could let us work more on preventive measures to decrease risk factors. Early identification, lifestyle modification, adherence and compliance to gluten-free diet, could slow the risk of AF in those patients with CD. This work was presented as an abstract (https://eventscribe.com/2018/ACG/ajaxcalls/PosterInfo.asp?PosterID=161644&efp=RFNSWFFHSFY2NDI0&rnd=3.819197E-02)

## Conclusions

A significant association between celiac disease and risk of atrial fibrillation was reported in this study. There is a 38% increased risk of atrial fibrillation in patients with celiac disease as compared to individuals without celiac disease used as controls. Additional studies are needed to clarify the mechanistic link between atrial fibrillation and celiac disease.
